# VISTA deficiency attenuates antibody-induced arthritis and alters macrophage gene expression in response to simulated immune complexes

**DOI:** 10.1186/s13075-017-1474-y

**Published:** 2017-12-08

**Authors:** Sabrina Ceeraz, Susan K. Eszterhas, Petra A. Sergent, David A. Armstrong, Alix Ashare, Thomas Broughton, Li Wang, Dov Pechenick, Christopher M. Burns, Randolph J. Noelle, Matthew P. Vincenti, Roy A. Fava

**Affiliations:** 1 Department of Microbiology and Immunology, Geisel School of Medicine at Dartmouth, Lebanon, NH 03756 USA; 2 Norris Cotton Cancer Center, Dartmouth Hitchcock Medical Center, Lebanon, NH 03756 USA; 3 Department of Veterans Affairs, Research Service, White River Junction, VT 05009 USA; 4 Pulmonary and Critical Care Medicine, Dartmouth-Hitchcock Medical Center, Lebanon, NH USA; 5 Microbiology and Immunology & Cancer Center Medical College of Wisconsin, Milwaukee, WI USA; 6 ImmuNext INC, 1 Medical Center Drive, Lebanon, NH 03756 USA; 7 Department of Medicine, Section of Rheumatology, Geisel School of Medicine at Dartmouth, Dartmouth-Hitchcock Medical Center, 1 Medical Center Drive, Lebanon, NH 03756 USA; 8 Department of Medicine, Geisel School of Medicine at Dartmouth, Dartmouth Hitchcock Medical Center, Lebanon, NH 03756 USA

**Keywords:** Arthritis, Macrophages, Antigen–antibody complex, Autoimmunity, Receptors, Complement

## Abstract

**Background:**

In addition to activated T cells, the immune checkpoint inhibitor “V domain-containing Ig suppressor of T-cell activation” (VISTA) is expressed by myeloid cell types, including macrophages and neutrophils. The importance of VISTA expression by myeloid cells to antibody-induced arthritis and its potential for relevance in human disease was evaluated.

**Methods:**

VISTA was immunolocalized in normal and arthritic human synovial tissue sections and synovial tissue lysates were subjected to western blot analysis. The collagen antibody-induced arthritis model (CAIA) was performed with DBA/1 J mice treated with antibodies against VISTA and with VISTA-deficient mice (V-KO). Total mRNA from arthritic joints, spleens, and cultured macrophages was analyzed with NanoString arrays. Cytokines secreted by splenic inflammatory macrophages were determined. In-vitro chemotaxis and signal transduction assays were performed with cultured macrophages.

**Results:**

VISTA protein was localized to synovial membrane cells, neutrophils, and scattered cells in lymphocyte-rich foci and was detected by western blot analysis in normal synovium and synovium from rheumatoid arthritis patients. Deficiency of VISTA or treatment of mice with anti-VISTA monoclonal antibodies attenuated CAIA. Joint damage and MMP-3 expression were significantly reduced in V-KO mice. Surface expression of C5a receptor was reduced on monocytes, neutrophils, and cultured macrophages from V-KO. Upon Fc receptor engagement in vitro, gene expression by V-KO macrophages was altered profoundly compared to WT, including a significant induction of IL-1 receptor antagonist (IL1rn).

**Conclusions:**

VISTA expression supports immune-complex inflammation in CAIA and VISTA is expressed in human synovium. VISTA supports optimal responses to C5a and modulates macrophage responses to immune complexes.

**Electronic supplementary material:**

The online version of this article (doi:10.1186/s13075-017-1474-y) contains supplementary material, which is available to authorized users.

## Background

Rheumatoid arthritis (RA) is a debilitating autoimmune disease, involving many cell types that drive chronic inflammation resulting in destruction of cartilage, tendon, and bone in articular joints [1, 2]. Current therapies can slow disease progression in many patients, but not all [3]. There therefore remains a need for identification of new therapeutic targets in RA.

V domain-containing Ig suppressor of T-cell activation (VISTA) is expressed predominantly by myeloid cells and T lymphocytes, and can mediate signaling between T cells and antigen-presenting cells (APC) [4, 5]. The murine protein is termed VISTA (also Dies1 or PD-1H) and the corresponding human protein is termed C10orf54 (also B7-H5, GI24, SISP1, or PP2135) [4]. VISTA can suppress T-cell-mediated immune responses in mouse models of liver disease, neurodegenerative disease, and cancer [6–14]. Moreover, studies in experimental autoimmune encephalomyelitis (EAE) [14] and murine lupus [15] revealed that VISTA expression by APC and myeloid subsets is equally important to its expression by T lymphocytes. Thus VISTA can modulate disease models through both its expression on T lymphocytes and on myeloid-derived cells. However, since neither a specific receptor for VISTA nor an intrinsic signaling capability has been identified, the precise biochemical mechanism by which VISTA mediates its biologic effects is not completely understood.

The regulation of VISTA and the significance of VISTA expression on monocytes/macrophages have only just begun to be explored. VISTA can be detected by flow cytometry on freshly isolated human monocytes, but its expression is lost when monocytes are cultured in vitro [14]. VISTA protein was increased on human monocytes by infection with HIV, and its induction promoted inflammatory cytokine gene expression [16] suggesting a link between VISTA expression and gene expression in monocytes. CD68-positive macrophages that expressed VISTA were observed in human tumors in vivo during a clinical trial with anti-CTLA-4, but the factors that regulate VISTA expression in macrophages remain unclear [17].

To investigate the role of VISTA on myeloid-derived cells in arthritis we chose the collagen antibody-induced arthritis (CAIA) model since monocytes and neutrophils are essential for this model, while T and B lymphocytes are not required. Thus CAIA is ideal to investigate the role of VISTA in “innate” joint inflammation driven solely by phagocyte responses to immune complexes and complement activation [18, 19]. The term “innate” inflammation here refers to proinflammatory responses driven by the engagement of complement receptors, chemokine receptors, and Fc receptors on phagocytes. CAIA is critically dependent upon such phagocyte responses [20]. Mice deficient in Fc-RIII and responses to C5a are especially resistant to CAIA induction due to impaired activation and recruitment of phagocytes [21].

Here we show that the human analog of murine VISTA is present in cells in human synovium. We also show that VISTA expression on myeloid cells is essential for optimal induction of arthritis in the CAIA model. Our data suggest that VISTA may contribute to inflammatory arthritis, in part through modulating phagocyte responses to C5a and in part by modulating responses of macrophages triggered by Fc receptor binding of immune complexes. The biochemical basis for these findings awaits the discovery of VISTA binding partners and signaling pathways.

## Methods

### Mice

DBA/1 J mice were purchased from Jackson Labs. VISTA-deficient (V-KO) mice were produced on the C57Bl/6 genetic background as described previously [13]. V-KO mice were bred at the VA Medical Center. All procedures were approved by the VA Medical Center IACUC, and comply with federal guidelines.

### CAIA model

Hamster anti-mouse VISTA antibody MH5A was purchased from Biolegend. Anti-mouse VISTA antibody (8G8) was produced in hamster by the Noelle Lab at Geisel Medical School. The specificity of 8G8 was confirmed using VISTA-specific ELISAs, assessing the distribution of antigen across cell lineages and the absence of detection on cells from V-KO mice. CAIA was induced in DBA/1 J mice by IP injection of 1.5 mg 5-clone Cocktail (Chondrex, Redmond, WA, USA) with an IP injection of 50 μg LPS given 3 days afterward. Mice were treated by IP injections of monoclonal antibodies (100 μg MH5A or 300 μg 8G8) or control hamster IgG beginning 3 days before induction of CAIA and every 3 days thereafter. CAIA was induced in V-KO and C57Bl/6 WT mice by IP injection of 5 mg of 5-clone Cocktail (Chondrex) and an IP injection of 50 μg LPS given 3 days afterward. Animals were scored daily for inflammation in each paw using a scale of 0–4; the total scores for each mouse were summed and the mean of these sums for each experimental group was plotted as the arthritis score with standard error and statistical analysis by nonparametric Mann–Whitney test. The data shown are representative of multiple experiments performed with V-KO mice and antibody treatment.

Histologic assessment of joint damage was performed using tissue sections of formalin-fixed decalcified joints of mice (knees, ankles, paws) collected 13 days after initiation of CAIA. Histologic analyses in preliminary experiments indicated that arthritis initially develops in the knee joints in wild-type mice several days prior to the appearance of overt paw/ankle swelling; consequently, knee joints were scored and analyzed. A cumulative scoring method was used since many distal joints of V-KO mice were found to be completely devoid of signs of arthritis, making the calculation of mean scores and statistical analyses for the experimental groups particularly difficult. Three tissue sections representing 500 μm of tissue at the approximate center of joints were examined and scored (0–4) for extent of pannus formation, cartilage damage/loss, and bone erosions.

### Cell culture

Bone marrow-derived macrophage (BMDM) cultures were prepared from V-KO and C57Bl/6 (WT) mice as described previously [22]. Briefly, whole bone marrow was flushed from the femurs and cultured in BMDM medium (RPMI supplemented with 10% FBS, 50 ng/ml recombinant mouse CSF-1, and Pen/Strep). Cultures were fed on day 3 and then harvested on days 5 and 7 for total cell protein and RNA. Splenocytes were harvested from 7-week-old male C57/Bl/6 and V-KO mice and inflammatory monocytes, F4/80^+^Gr1^+^CD11b^+^ cells expressing C5aR, were sorted on a BD FACSAria III (BD Bioscience). Cells were stimulated with 10 ng/ml LPS (Sigma-Aldrich) for 24 hours and supernatants were collected and screened for cytokines/chemokines by Luminex assay.

### Localization of VISTA in synovium

Localization of VISTA was performed on tissue sections of formalin-fixed and paraffin-embedded human synovial tissue selected from a collection of de-identified, archived materials (RAF) or were purchased from a commercial source (Origene, Rockville, MD, USA). Tissue sections were deparaffinized and subjected to antigen retrieval with citrate buffer, and endogenous peroxidase was quenched with hydrogen-peroxide in methanol. Nonspecific binding was blocked with 2.5% goat serum and slides were incubated at 4 °C with rabbit monoclonal anti-VISTA (catalog number D1L2G XP; Cell Signaling), without primary antibody or with an equal concentration of isotype control (catalog number SP137; Abcam). Signal amplification was performed with Biotin-XX Tyramide SuperBoost (catalog number B40921; Invitrogen) followed by reaction with streptavidin horseradish peroxidase (catalog number S911; Invitrogen) and insoluble substrate development with diamino-benzidine (ImmPact DAB, catalog number SK-4103; Vector Laboratories). Slides were counterstained with Hematoxylin2 (catalog number 7231; ThermoFisher).

### Western blot analysis

Whole cell extracts from BMDMs were harvested in 200 ml of 2× Sample Buffer (Sigma Chemical, St. Louis, MO, USA) and heated to 95 °C for 5 minutes. Twenty microliters of each sample were resolved on 10% SDS-PAGE gels and transferred to Immobilon-P membranes (Millipore, Inc., Billerica, MA, USA). Blots were probed with antibodies specific for mouse VISTA (R&D Biosystems, Minneapolis, MN, USA), or with antibodies for phosphorylated AKT, total AKT, phosphorylated ERK, total ERK, beta actin or tubulin (Cell Signaling Technology, Danvers, MA, USA) and were visualized by chemiluminescence.

### Flow cytometric analysis

Splenocytes were incubated with Tris-buffered ammonium chloride (ACT; Sigma-Aldrich) to lyse red blood cells. Cells were then washed in PBS for 5 minutes at 1500 rpm followed by staining with live/dead Fixable Dead Cell Stain (Life Technologies) at RT for 20 minutes. Cells were subsequently washed twice in PBS for 5 minutes at 1500 rpm at 4 °C and stained with the appropriate directly labeled antibodies on ice for 20 minutes. Cells were then washed twice in PBS for 5 minutes at 1500 rpm at 4 °C and fixed until acquisition. Samples were run on the MACSQuant Analyzer 10 (Miltenyi Biotec) and analyzed using the FlowJo software (Treestar). For whole bone marrow, BMDM cultures, purified neutrophils, and PBMC were analyzed by flow cytometry using a Becton Dickinson FACSCanto II cell analyzer and FACSDiva software. Fluorescently labeled antibodies against mouse VISTA (MH5A) C5aR, Cd11b, Ly6G, CD45, and F4/80 were purchased from BioLegend (San Diego, CA, USA), FcRI and FcRIV from BD Biosciences (San Jose, CA), FcRIII from R&D Systems (Minneapolis, MN), and FcRIIb from eBiosciences (ThermoFisher).

### Graphs and statistical analysis

Graphs were prepared using GraphPad Prism software versions 6 and 7 (GraphPad, San Diego, CA, USA). Statistical analysis is shown using the Student *t* test (two-tailed) at ***=*P* < 0.005, **=*P* < 0.025, and *=*P* < 0.05.

### Fc receptor activation of BMDMs by plate-bound IgG

Six-well tissue culture plates were coated overnight at 4 °C with the 5-clone monoclonal antibody cocktail containing IgG2a and IgG2b isotype monoclonal antibodies (100 μg/ml, 2 ml/well). Wells were washed twice with PBS prior to the addition of cells. BMDMs from WT and V-KO mice were cultured for 6 days on nontreated tissue culture plates and then harvested by incubating for 10 minutes at 4 °C and scraping with a Cell Lifter (Thermo-Fisher Scientific). Cells were washed, counted, and then added to either coated or uncoated wells at 3 × 10^5^ cells/well. Following an additional 24 hours of culture, RNA was isolated using the RNeasy Minikit (Qiagen, Germantown, MD, USA) for analysis by NanoString assay.

### NanoString assay

RNA was isolated from the frozen spleen tissue blocks using the PureLink RNA Mini Kit (Ambion/Invitrogen) and PureLink DNase Set (Ambion/Invitrogen). To isolate RNA samples from formalin-fixed paraffin-embedded knee joints, the Qiagen RNeasy FFPE Kit (Qiagen) was used. All samples were run on a Bioanalyzer to determine purity. Gene expression was measured using the nCounter^®^ GX Mouse Immunology, Mouse Inflammation, and Mouse Myeloid Cell codesets (NanoString Technologies), run and read on an nCounter^®^ Analysis System (NanoString Technologies).

To analyze the NanoString data, gene expression data from NanoString were normalized in nSolver and log_2_-transformed for further analysis for differential expression. Data from joint samples were analyzed in R using unpaired *t* tests followed by Benjamini and Hochberg multiple hypothesis correction. Data from spleen samples were analyzed in R/Bioconductor using the limma package followed by Benjamini and Hochberg multiple hypothesis correction. Boxplots were made using the R package ggplot2. Heat maps were constructed by UPGMA hierarchical clustering of gene expression using 1 – Pearson’s correlation coefficient as the distance, followed by *Z*-score row normalization for plotting in Excel or the gplots package in R [23–27]. Gene expression values from immune-complex-stimulated BMDM cultures were normalized to the geometric mean of housekeeping genes using nSolver software (NanoString Technologies). Gene expression values between V-KO and WT BMDM cultures were compared by multiple two-tailed *t* tests and discoveries were identified by the Benjamini and Hochberg method, with a *Q* value of 1% (GraphPad Prism 7). Discovered genes that showed at least a 2-fold change between V-KO and WT BMDM cultures, either under basal or IgG-stimulated conditions, were chosen for hierarchical clustering. A heat map was generated using nSolver software, with a Genes *z*-score transformation, average linkage, and Pearson’s correlation as the distance metric.

### Migration assay

BMDMs in complete media were plated in the upper wells of invasion chambers fitted with 8-μM FluoroBlok inserts (BD Biosciences, Billerica, MA, USA) according to the manufacturer’s instructions. Medium containing 50 ng/ml C5a was added to the lower chamber to act as a chemoattractant. After an additional 2 hours, invaded cells were stained with 4 μM Calcein AM and imaged using a Nikon ECLIPSE TS100 inverted microscope fitted with 4× objective and a Spot digital camera. Migrated cells were counted in triplicate wells using NIS Elements Advanced Research imaging software.

## Results

### VISTA protein is present in human synovium

**Fig. 1:**
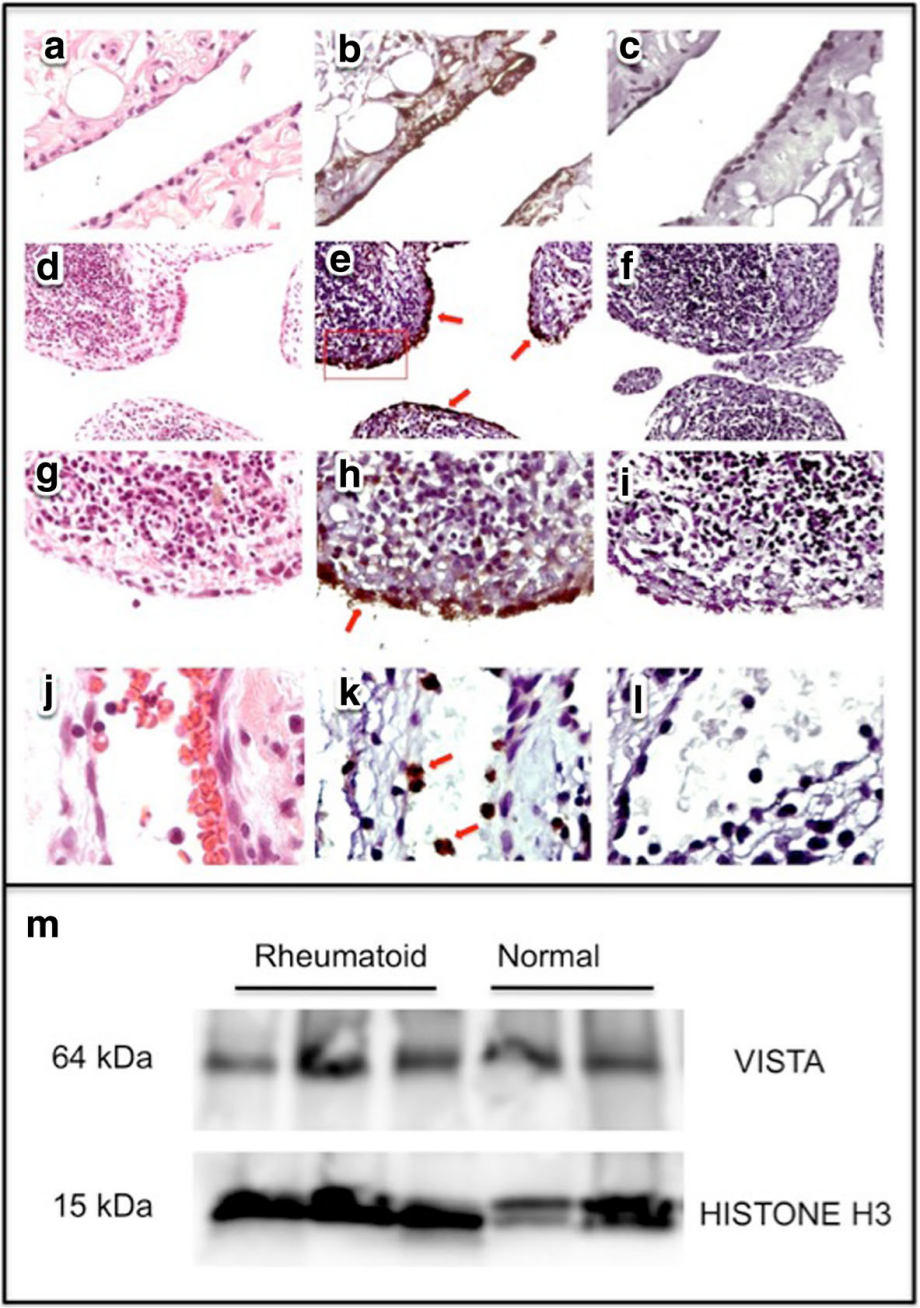
Immunoreactive VISTA is detected in human synovium tissue sections and lysates of normal and rheumatoid human synovium. **a**–**c** Normal human synovium. **d**–**l** RA synovium. **a**, **d**, **g**, **j** Hematoxylin-and-eosin-stained paraffin tissue sections. **b**, **e**, **h**, **k** Anti-VISTA peroxidase staining developed with diamino-benzidine. **e** Arrows denote synovial membrane staining; rectangle encloses area enlarged in (**g**–**i**). **c**, **f**, **i**, **l** Nonspecific isotype control antibody staining developed with diamino-benzidine. **j**–**l** Neutrophils in vessel in synovium; arrows denote VISTA-stained neutrophils in (**k**). **m** Western blot of synovial tissue lysates from rheumatoid arthritis patients (*n* = 3) and normal synovium (*n* = 2) stained with anti-human VISTA antibody or anti-histone H3 as sample loading control. VISTA; V domain-containing Ig suppressor of T-cell activation

A procedure for localization of immunoreactive VISTA was developed and optimized by preliminary staining of sections of human tonsil in which VISTA-expressing cells were stained predominately in the interfollicular zone in a pattern similar to that of CD68-expressing macrophages/dendritic cells (data not shown). In human synovium, the highest expression of VISTA protein was by the synovial lining membrane cells. This was the case in both normal human synovium (Fig. 1b) and synovium of patients with RA (Fig. 1e, h). Virtually every cell of the synovial lining reacted strongly with anti-VISTA (Fig. 1b, e, h) but not with isotype control antibody (Fig. 1c, f, i). Also, cells identified by their polymorphic nuclei as neutrophils were strongly stained for VISTA immunoreactivity (Fig. 1k). No staining occurred in controls performed with nonspecific IgG of the same isotype (Fig. 1l), ensuring that nonspecific IgG binding or staining due to unquenched endogenous peroxidase activity was not a confounding factor. A few cells present in lymphoid follicles from a subset of synovial tissue specimens stained for VISTA, although the majority of cells were not stained (Fig. 1h). Note that it is possible for expression of VISTA protein by some cell types in the synovium to be below the limits of detection by this method.

VISTA was also detected by western blot analyses of total protein lysates of synovium of RA patients (*n* = 3) as well as normal synovium (*n* = 2) (Fig. 1m), confirming by a second method that VISTA is present in cells in normal synovium and in the inflamed synovium of patients with RA as well. The significance of VISTA expression by resident cells of synovium to the pathogenesis of arthritis remains unknown.

### Systemic treatment with anti-VISTA and VISTA deficiency attenuates CAIA

**Fig. 2:**
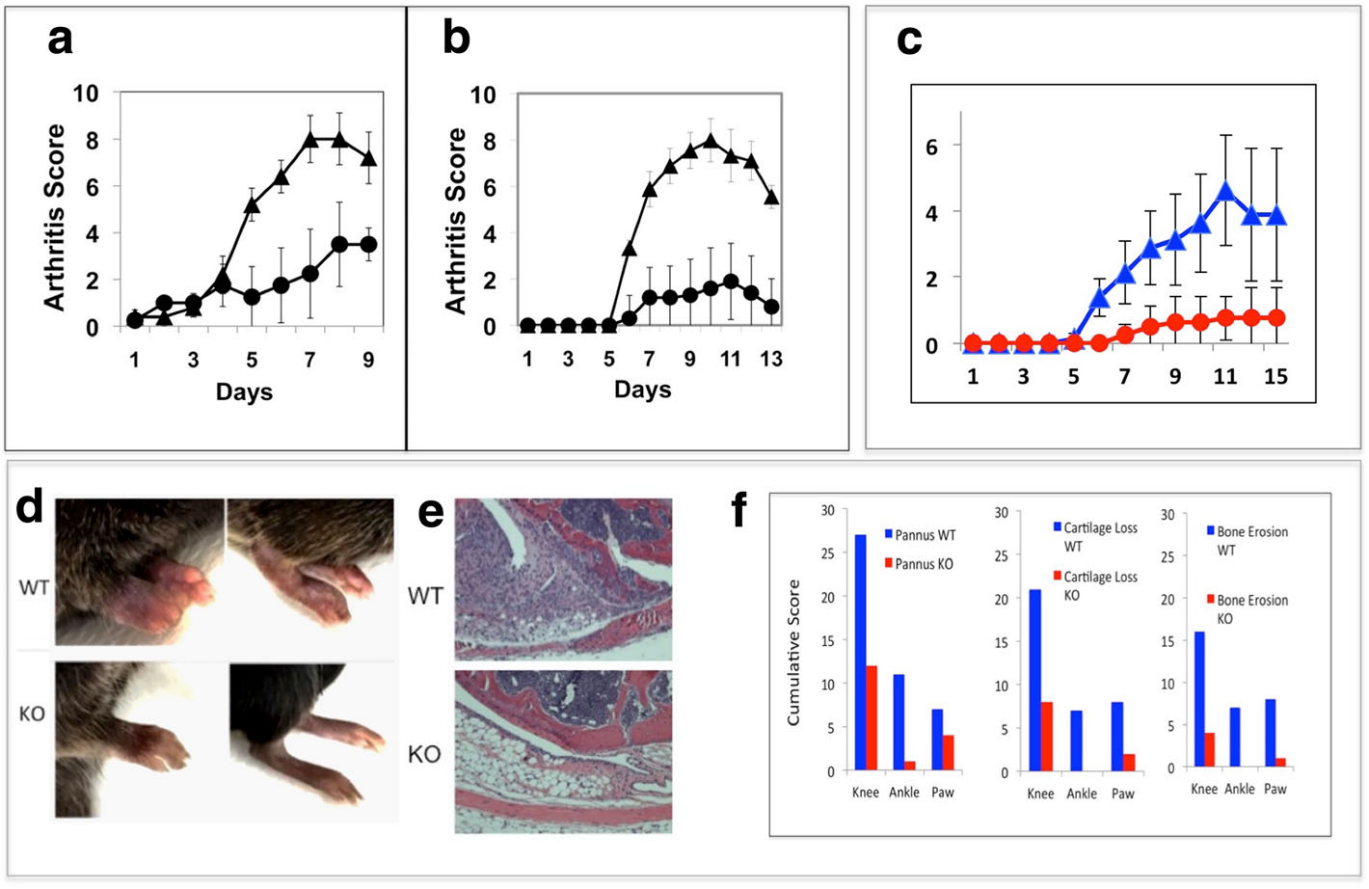
Antibodies against VISTA and VISTA deficiency protect mice from collagen antibody-induced arthritis (CAIA). (**a**, **b**) DBA/1 J mice or (**c**–**f**) C57Bl/6 wild-type (WT) or V-KO (KO) mice were subjected to the CAIA model (*n* = 6 per group). **a** Mean arthritis scores of mice treated with anti-VISTA clone MH5A (circles) or hamster Ig (triangles). **b** Mean arthritis scores of mice treated with anti-VISTA clone 8G8 (circles) or hamster IgG (triangles). **c** Mean arthritis scores for WT mice (blue triangles) and V-KO mice (red circles). **d** Representative hind paws of WT and KO mice on day 13. **e** Representative H&E-stained tissue sections of the central aspect of knee joints from WT and KO mice on day 13. **f** Cumulative histology scores for pannus, cartilage loss, and bone erosions for knee, ankle, and paw tissue sections from WT mice (blue bars) and V-KO mice (red bars) on day 13

DBA/1 J mice were treated with either nonspecific hamster IgG or one of two different anti-VISTA mAbs; mAb clone MH5A (Fig. 2a) or mAb clone 8G8 (Fig. 2b). With each of the antibodies, prophylactic treatment of mice with systemic anti-VISTA monoclonal antibody greatly reduced paw-swelling arthritis scores (Fig. 2a, b). CAIA was also performed using mice deficient in VISTA gene expression, V-KO mice, to verify that the inhibition of CAIA by treatment of DBA/1 J mice with anti-mouse VISTA antibodies was due to specific binding of antibodies to VISTA and not nonspecific effects. Compared to wild-type C57Bl/6 mice, V-KO mice were highly resistant to induction of arthritis and developed significantly lower arthritis scores and disease incidence (Fig. 2c). Examples of the swelling observed in paws of wild-type mice and a lack of overt paw swelling observed for many joints of V-KO mice are shown (Fig. 2d). Representative photomicrographs of the extensive pannus formation observed in knee joints of WT mice compared to minimal synovial pannus in knee joints of V-KO mice are also shown (Fig. 2e). Cumulative histologic scores for pannus formation, cartilage damage, and bone erosion were greater in WT mice than in V-KO mice in knees, ankles, and paws (Fig. 2f).

### Arthritic V-KO mice have reduced joint inflammation and tissue damage

**Fig. 3:**
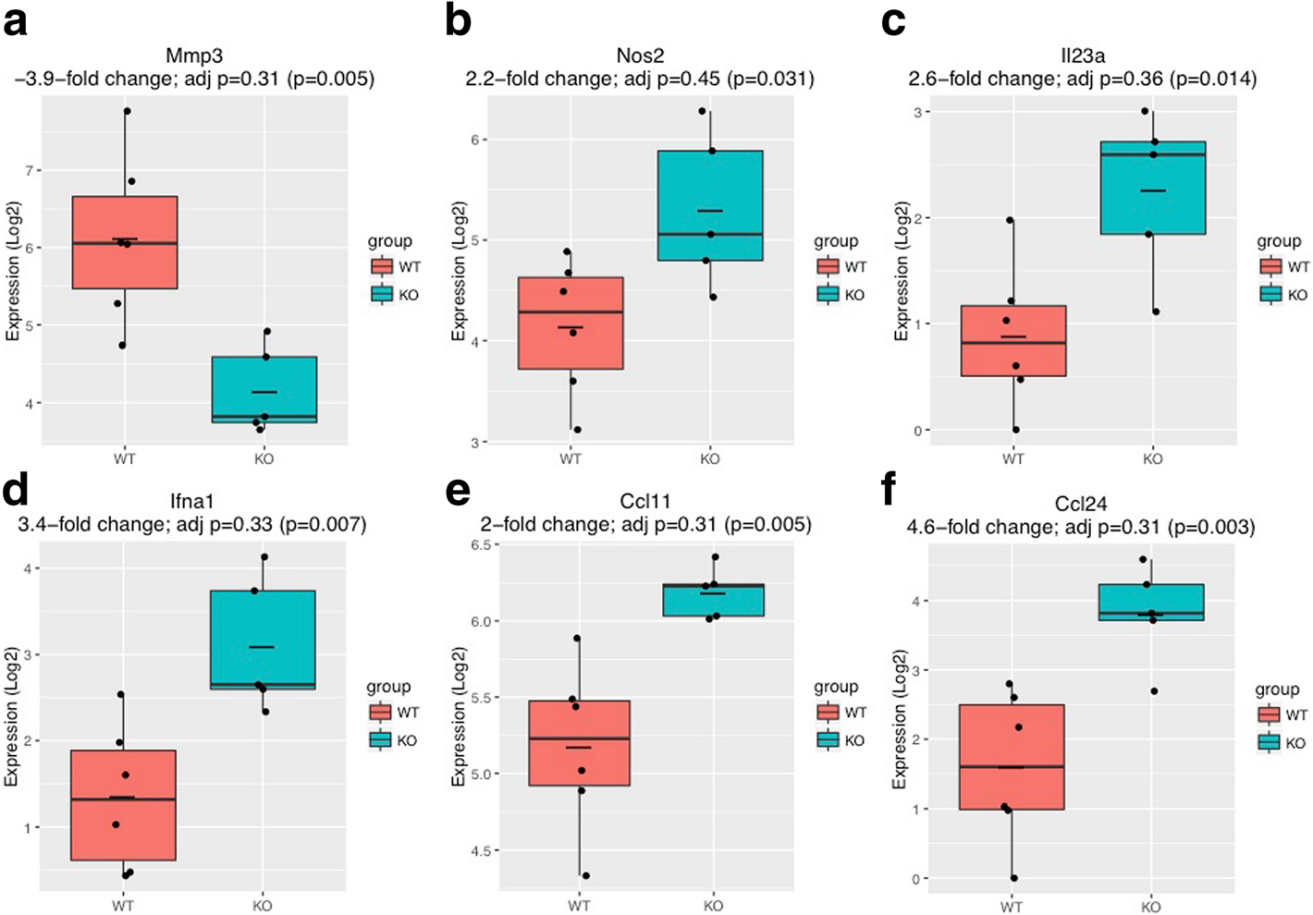
NanoString analysis of mRNA from arthritic joints of mice. CAIA was induced in wild-type (WT, *n* = 6) and V-KO (KO, *n* = 6) mice and mRNA was isolated from knee tissue on day 13 post induction. Each mRNA sample was analyzed using the NanoString nCounter^®^ Mouse Inflammation Kit. Groups of WT (red) versus V-KO (blue) were compared by unpaired *t* tests, with adjusted *p* values and raw *p* values shown in parentheses. adj adjusted. **a** Mmp3 (matrix metalloproteinase 3), **b** Nos2 (nitric oxide synthase 2), **c** Il23a (interleukin 23a), **d** Ifna (interferon alpha 1), **e** Ccl1 (C-C motif chemokine ligand 1), **f** Ccl24 (C-C motif chemokine ligand 24)

To further characterize the attenuation of joint inflammation in V-KO mice, we isolated RNA from knee joints at day 13 of CAIA and performed a gene profiling analysis using the NanoString nCounter^®^ Mouse Inflammation Kit. Knee joints were chosen for this analysis because histologic analyses showed that the incidence of inflammation in knees was more uniform for V-KO mice and WT mice than it was in paws (inflammation in knees actually precedes inflammation in distal joints by several days), since many V-KO mice completely lacked inflammation of paws and digits. One noteworthy finding was that the knee joints of V-KO mice had nearly 4-fold less MMP-3 gene expression compared to their WT counterparts on day 13 of arthritis (Fig. 3), consistent with the reduced cartilage and bone erosion scores (Fig. 2f). Interestingly, MMP-3 expression was reduced in V-KO joints even though IL-1B, IL-6, or TNF was not reduced at this point of disease (data not shown), suggesting that MMP-3 expression in joints may not have been regulated primarily by inflammatory cytokines, as is often the case in the much less complex cell culture model systems. Surprisingly, certain genes implicated in arthritis pathogenesis were more than 2-fold higher in arthritic V-KO joints, including NOS2, IL-23a, and IFNA1 (Fig. 3). Among the genes that were overexpressed in V-KO arthritic joints were several typically associated with the M2 macrophage phenotype, usually associated with resolution or antagonism of inflammation, including CCL11 and CCL24 (Fig. 3) (a comprehensive gene list is included in Additional file 1: Figure S1).

Using NanoString technology, gene expression analysis of spleens from WT and V-KO mice undergoing CAIA was performed. This analysis of total splenocytes revealed significant reductions in genes associated with macrophage function, including CD163, CD36, Cd1d1, and CD14 in spleens from V-KO mice (Additional file 2: Figure S2).

### Macrophages cultured from V-KO mice have reduced rapid responses to C5a in vitro

Since phagocyte responses to the complement-derived peptide C5a are critical for induction of the CAIA model, we investigated the plasma concentrations of C5a during CAIA induction, the expression of the cell surface C5a receptor, and selected in-vitro responses to C5a for WT versus V-KO mice [21]. Comparable levels of C5a were detected in the plasma of WT and V-KO mice on day 6 after CAIA initiation, rendering it unlikely that attenuated induction of disease in V-KO mice was due to defective generation of complement fragment C5a (data not shown).

**Fig. 4:**
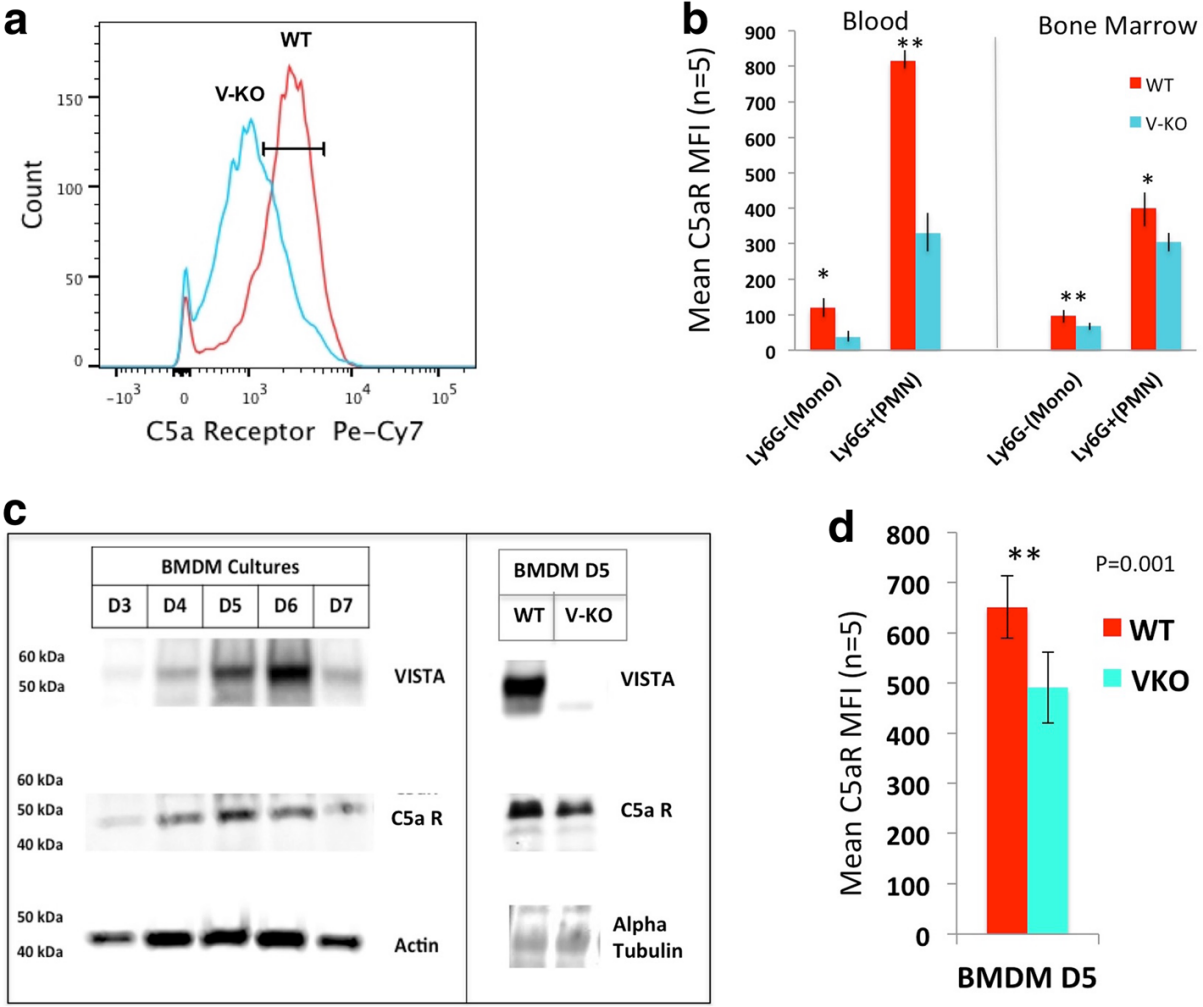
Reduced cell surface C5a receptor expression on blood and bone marrow monocytes, on neutrophils, and on cultured BMDMs in V-KO mice. **a** Representative histogram of cell surface expression of C5aR on BMDMs from wild-type (WT) and V-KO (V-KO) mice (culture day 5). **b** FACS of cell surface C5aR expression for cd11b^+^/Ly6G^–^ monocytes (mono) and cd11b^+^/Ly6G^+^ neutrophils (PMN) in peripheral blood and bone marrow of WT and V-KO mice. **c** Western blot analyses of BMDM cells cultured from WT and V-KO mice on days 3–7 of culture (left) and for day 5 BMDMs (right). **d** Mean fluorescence intensity (MFI) determined by FACS analysis of cell surface expression of C5aR on day 5 cultured BMDMs from WT (red bars) and V-KO (green bars) mice (*n* = 6). Student *t* test (two-tailed): **p < 0.025; *p < 0.05

Interestingly, FACS analysis of neutrophils and monocytes showed that cell surface expression of C5a receptor was consistently reduced for V-KO mice compared to cells from WT mice, both on cells in the peripheral blood and on cells in the bone marrow (Fig. 4a, b). This difference in MFI for WT versus V-KO cell surface expression of C5aR was statistically significant both in vivo and in cultured BMDMs (Fig. 4b, d). Reduced C5a receptor was also observed in a monocyte subset of particular interest in joint inflammation, the F4/80^+^/Gr1^+^/CD11b^+^ “inflammatory” monocyte (Additional file 3: Figure S3), which was further examined. Inflammatory monocytes that expressed C5aR were reduced in abundance in spleens of V-KO mice compared to WT mice and this subset also had reduced cell surface C5aR expression as evidenced by a difference in mean MFI values in FACS analyses (WT (*n* = 6) MFI = 1192 ± 212; V-KO (*n* = 6) MFI = 428 ± 76.3; *p* = 007). The inflammatory monocyte subset was isolated by FACS analysis and cells were assayed for the production of inflammatory cytokines both with and without LPS stimulation. Cells from V-KO mice produced less TNF, IL-17, and IL-12p70 in response to LPS compared to cells from WT mice (Additional file 3: Figure S3). These results indicate that the inflammatory subset of monocytes in V-KO mice has important functional differences that could contribute to the attenuation of CAIA.

To further examine the role of VISTA on monocyte and macrophage function, BMDM cultures were established and the expression of VISTA protein was assessed during in-vitro differentiation of macrophages. VISTA was detected by western blot analysis as a 55-kDa protein with maximal expression observed on day 6 of culture of BMDMs from WT mice. The 55-kDa protein is the glycosylated form of VISTA [4]. This 55-kDa protein was completely absent from the lysates of V-KO BMDM cells as expected, as shown for day 5 of culture (Fig. 4c, right panel). Western blot analysis confirmed that other important macrophage proteins such as the C5a receptor were expressed in BMDM cultures from WT and V-KO mice, and total cellular C5aR was expressed in similar amounts for WT and V-KO BMDMs as shown for day 5 of culture (Fig. 4c, right panel). The differentiation/maturation marker F4/80 was expressed similarly by both WT BMDM cells and V-KO BMDM cells when analyzed by flow cytometry, suggesting that, at least by some criteria, in-vitro monocyte differentiation in culture was not greatly altered by VISTA deficiency. However, cell surface expression C5aR was significantly reduced in V-KO BMDM cells (Fig. 4c). The reason for the reduced amount of C5a receptor on the surface of cells from V-KO mice is not yet understood, since total protein levels for C5aR in WT and V-KO BMDM cultures assessed by semi-quantitative western blot analysis of whole cell extracts appeared comparable (Fig. 4c, right panel). Thus, although the underlying mechanism is not understood, C5aR surface expression was reduced in splenic inflammatory monocytes, cultured BMDMs, peripheral blood monocytes, and neutrophils of V-KO mice, possibly contributing to the attenuation of CAIA in these mice.

**Fig. 5:**
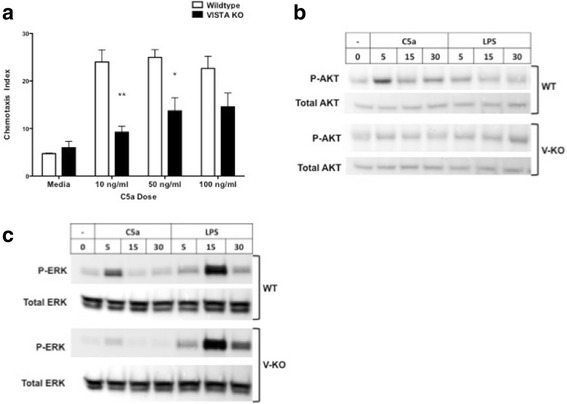
Reduced chemotactic and signal transduction responses to C5a in BMDM cells from V-KO mice. **a** Chemotactic response to C5a by BMDM cells cultured from wild-type (WT) mice (open bars) and V-KO (KO) mice (black bars). **b** Western blot analysis of total and phosphorylated AKT (AKT pathway activation) in WT and V-KO BMDMs stimulated with 50 ng/ml C5a for indicated time (minutes). **c** Western blot analysis for total and phosphorylated ERK (MAPK pathway activation) in WT and V-KO BMDMs stimulated with 50 ng/ml C5a for indicated time (minutes). Student *t* test (two-tailed): **p < 0.025; *p < 0.05. LPS lipopolysaccharide

The effect of the reduction in C5aR expression on the surface of BMDMs upon chemotactic responses to C5a in vitro was examined. Compared to BMDMs from WT mice, BMDMs from V-KO mice exhibited a significantly reduced chemotactic response to C5a at doses of 10 and 50 ng/ml (Fig. 5a). To determine whether C5a receptor signal transduction was also affected in BMDMs from V-KO mice, we assayed the phosphorylation of AKT and ERK, two members of protein kinase signaling pathways normally activated very rapidly by C5a [28]. AKT phosphorylation was maximally induced within 5 minutes by C5a in BMDMs from WT mice, while phosphorylation of AKT was reduced in BMDMs from V-KO mice (Fig. 5b). As expected, AKT was not rapidly phosphorylated by LPS stimulation in WT cells, or in V-KO cells. C5a also induced robust phosphorylation of ERK in WT BMDMs as expected, while ERK phosphorylation was attenuated in V-KO cells (Fig. 5c). In contrast to C5a, LPS activation of ERK phosphorylation was comparable in BMDMs from the two strains. Taken together, the reduced basal cell-surface expression of C5aR in vivo and the reduced responses to C5a observed in cultured cells from V-KO mice suggest the possibility that altered C5aR responses contribute to the attenuation of CAIA in V-KO mice, although not yet definitively demonstrated in vivo.

### Heightened anti-inflammatory gene expression pattern of V-KO macrophages upon Fc receptor engagement

**Fig. 6:**
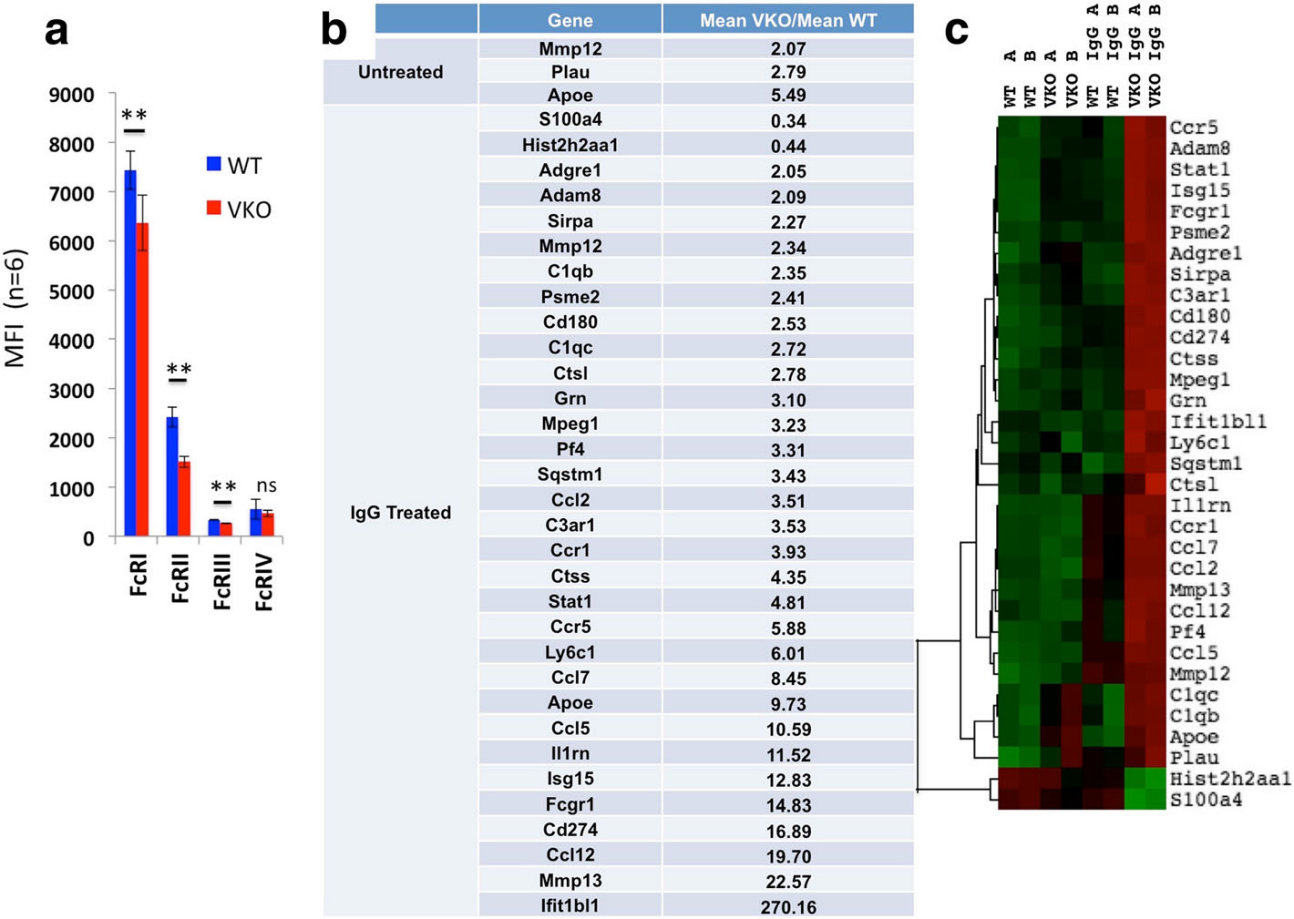
Altered gene expression by BMDMs from V-KO mice induced by Fc receptor engagement. BMDMs from wild-type (WT, *n* = 2) and V-KO (KO, *n* = 2) mice were plated onto culture plates coated with IgG2a and IgG2b isotype monoclonal antibodies (5-clone Cocktail) or on uncoated plates (untreated). Total mRNA was isolated after 24 hours of culture and analyzed by NanoString using the Mouse Myeloid codeset. **a** Average mean fluorescence intensity (MFI) values for Fc receptors (I, IIb, III, IV) as determined by FACS analysis of BMDMs. (**b**) Genes that changed more than 2-fold with adjusted *p* < 0.01 shown for untreated (top) and IgG-treated (bottom) cells. **c** Clustering analysis of genes indicated in (**b**). Replicate macrophage cultures were prepared from two individual mice of each type (i.e., WT-A, WT-B, VKO-A, VKO-B) and mRNA analyzed individually

Immune complexes generated in the CAIA model trigger myeloid cell responses through Fc gamma receptor signaling (Fcgr) [29]. Using the NanoString Myeloid Cell codeset, we performed NanoString gene expression analysis on WT and V-KO BMDMs exposed to plate-bound Chondrex 5-clone antibodies (a mixture of IgG2a and IgG2b antibodies) to simulate the type of immune complex binding to Fc receptors that occurs in CAIA [30]. FACS analysis revealed small but significant reductions in the levels of cell surface expression of FcRI, FcRII, and FcRIII on V-KO cultured macrophages used for this analysis, while there was no difference in FcRIV expression (Fig. 6a). Unstimulated V-KO and WT BMDMs displayed very similar basal gene expression; however, after stimulation with plate-bound IgG, the gene expression \pattern differed greatly for V-KO macrophages and WT macrophages. In fact, 32 genes were differentially expressed between V-KO BMDMs and WT BMDMs after they were stimulated by plate-bound simulated IgG immune complexes (Fig. 6b, c), several more than 10-fold. By comparison, only three genes were differentially expressed by the unstimulated BMDMs. These differences were detected with a 1% false discovery rate (Benjamini and Hochberg) and focusing on 2-fold changes or greater. For example, IgG-stimulated V-KO BMDMs had much higher levels of interleukin-1 receptor antagonist (Il1rn), Mmp13, Fcgr1, and Ifit1bl1 mRNA compared to IgG-treated WT BMDMs. An ELISA performed for IL-1 receptor antagonist confirmed that the protein was made and secreted into culture medium (data not shown). In contrast to induced genes, IgG-treated V-KO BMDMs had less than half the levels of S100a4 and Hist2h2aa1 mRNA observed in the WT IgG-treated cultures. These data demonstrate that V-KO macrophages have very different gene expression responses to simulated IgG immune complexes in vitro, and these differences are likely to have contributed to the overall attenuation of inflammation in V-KO mice that was observed in the CAIA model.

## Discussion

VISTA is an immune checkpoint regulator protein that exerts profound effects in innate and adaptive immune disease mouse models such as experimental autoimmune encephalitis [13], lupus [15], and graft versus host disease [7]. Here we show for the first time that expression of VISTA by macrophages is instrumental in their responses to Fc receptor engagement. Also, we show that the human homolog of VISTA is expressed at the protein level in synovium and, moreover, that expression of VISTA by mouse myeloid-derived phagocytes is important to their ability to promote inflammation in arthritic joints in CAIA [20]. Thus, while VISTA has previously been shown repeatedly to be important in the context of cognate interactions that regulate T-cell actions, this is the first demonstration that VISTA expression by myeloid cells is required for optimal responses in inflammation driven by immune complexes. VISTA-deficient mice exhibited reduced paw swelling and were partially protected from the connective tissue degradation that results in CAIA. These effects were associated with attenuated myeloid cell responses to C5a and to a greatly altered pattern of gene expression in response to exposure of cultured macrophages to simulated immune complexes in the form of plate-bound IgG. The demonstration that suppression of inflammation in CAIA was also achieved by administration of anti-VISTA antibodies suggests a potential for use of similar antibodies to treat human rheumatoid arthritis.

Antibodies that recognize VISTA and the deletion of VISTA have elicited seemingly opposite effects in different disease models in previous studies. For example, deletion of VISTA exacerbated lupus [15] and anti-VISTA treatment exacerbated certain autoimmune models such as EAE [14] and skin inflammation [31]. On the other hand, anti-VISTA treatment completely prevented graft versus host disease [7]. The exact properties and epitopes involved with each monoclonal antibody against VISTA may account for these apparent differences. The antibodies used herein may have interfered with VISTA interactions with another unidentified protein(s) involved in mediating its biological effects, but currently the precise mechanism remains unknown. In the case of CAIA, the same antibody (MH5A) that blocked induction of GVH [7] greatly reduced joint inflammation, as did a second monoclonal antibody (8G8). The absence of any major role for lymphocytes in induction of the CAIA model likely enabled the observation of “anti-inflammatory” consequences of VISTA deficiency. It has been reported that T or B lymphocytes may be able to impact CAIA in both a positive and a negative sense, through regulatory capacities such as the release of proinflammatory cytokines by Th17 cells, or alternatively by the release of suppressive cytokines such as IL-10 and IL-4 from T-regulatory cells, when C-II reactive T cells or T-regulatory cells are adoptively transferred in CAIA [32, 33]. We found that mRNA for suppressive cytokines TGF-β, IL-10, or IL-4 was not highly elevated in NanoString analysis of joints from V-KO mice, suggesting that enhanced activation of T-regulatory cells by VISTA deficiency was not a major factor in attenuation of CAIA in V-KO mice. Moreover, recent data have shown that VISTA-deficient mice have a reduced pool size of induced T-regs, due to a role for VISTA in the differentiation and stability of this regulatory T-lymphocyte subset which might counteract the putative activation of T-regs by VISTA blockade or deficiency [34].

Increasing evidence is now appearing that VISTA is involved in fundamental or “innate” responses of myeloid cells that are important components of its biology. A very recent report revealed that VISTA is involved in myeloid cell responses to TLR7 [31]. Here we identified two other “innate response” pathways of myeloid cells (C5a and Fc receptors) that are altered in the absence of VISTA or when certain anti-VISTA antibodies bind their antigen systemically. It is not yet precisely clear which pathways were stimulated by adhering BMDMs onto plates coated with the mixture of mouse monoclonal antibodies of the IgG2a and IgG2b isotypes in this study. Both “activating” and “inhibitory” Fc receptors may bind these isotypes with varying affinities (IgG2a, FcRI > > FcRIV > FcRIII/FcRIIb; IgG2b, FcRIV > > FcRIIb > FcRIII) and it is well known that the overall balance of signaling pathways initiated by immune-complex binding to Fc receptors determines the functional phenotype of the responding cell [35]. What is clear is that the absence of VISTA greatly altered the gene expression pattern resulting from Fc receptor engagement. More work is required to define these pathways.

Protection of V-KO mice in the CAIA model was associated with nearly 4-fold less MMP-3 expression in arthritic joints compared to their WT counterparts. Others have shown that C5aR-deficient mice express less MMP-3 during CAIA [36], suggesting a possible connection between reduced C5aR surface expression in V-KO macrophages and reduced MMP-3 expression in the joints. In contrast to MMP-3, V-KO macrophages produced higher basal levels of MMP-12 mRNA and more MMP-13 mRNA in response to Fc receptor engagement. MMP-12 has been shown to dampen inflammation in arthritis by inactivating complement C3 and degrading neutrophil extracellular traps (NETS) [37]. Similarly, MMP-13 has anti-inflammatory effects through cleavage and inactivation of monocyte chemoattractant protein-3 [38]. In addition to these anti-inflammatory MMPs, V-KO macrophages had dramatically higher IL-1 receptor antagonist (Il1rn) expression in response to Fc receptor engagement compared to their WT counterparts. Indeed, IL-1 receptor antagonist or Anakinra (Kineret) is an FDA-approved treatment for human rheumatoid arthritis [39] and Il1rn deficiency in mice results in spontaneous polyarthropathies [40]. Thus, elevated Il1rn production by V-KO macrophages in response to immune complexes could attenuate the CAIA model, partly by antagonism of IL-1 induction of MMP-3 expression and its other effects on endothelium and chondrocytes. In contrast to Il1rn, the calcium binding protein S100a4 was downregulated in V-KO macrophages challenged with immune complexes. S100a4 expression is elevated in rheumatoid synovium and has been implicated as a mediator of synovial inflammation and pathogenesis through the upregulation of p53 target genes [41].

Many questions remain unanswered in this first report of a biological context in which VISTA deficiency has an inhibiting effect on inflammation associated with the autoimmune disease arthritis. This observation contrasts with a recent report showing that VISTA deficiency enhanced a murine lupus model [15]. It must be acknowledged, however, that although arthritis and lupus models share some biological pathways, such as a dependence on complement activation, formation of immune complexes, and Fc receptor activation, they are very different disease models. For example, disease develops very slowly in lupus models, over a period of months, in contrast to the rapid development of extensive inflammation in CAIA that occurs over a period of days. It seems likely that the gradual accumulation of changes in kidney tissue driven by the slow deposition of immune complexes in lupus models would result in different biological events compared to the very rapid and extensive activation of phagocyte response pathways in the CAIA model. Targeting of any anti-VISTA therapies to specific cell types, such as macrophages, may be required in complex diseases to take advantage of its role in supporting immune-complex inflammation. Discovery of precisely how on the molecular level VISTA modulates the range of responses reported for T lymphocytes and myeloid cells will be necessary to fully explain seemingly contradictory data sets.

## Conclusions

The data presented identify a role of the protein VISTA in myeloid phagocytic cells, particularly macrophages/monocytes, in responses to immune complexes and complement fragment C5a that may have relevance in arthritic pathology. VISTA is present on cells that reside in human synovium and may therefore represent a novel therapeutic target. More work is required to understand the underlying biochemical mechanism(s) by which these effects are mediated and whether or not VISTA (c10orf54) may represent a useful therapeutic target in human arthritis.

## Additional files

Additional file 1: Figure S1. showing gene profile of knee joints from WT and V-KO mice on day 13 of CAIA. RNA isolated from WT (*n* = 6) and KO (*n* = 5) paraffin-embedded joints. To examine gene expression, RNA was hybridized on the Mouse Inflammation NanoString plate and expression was read on an nCounter^®^ Analysis System. Each gene compared by unpaired *t* tests, raw *p* values presented alongside adjusted *p* values (see Methods) (JPG 166 kb)

Additional file 2: Figure S2. showing comparison of splenic gene expression profiles for arthritic and nonarthritic WT versus V-KO mice. RNA isolated from snap-frozen spleens taken from WT (*n* = 5), V-KO (*n* = 5), WT CAIA (*n* = 8), and V-KO CAIA (*n* = 8) mouse. To examine gene expression, RNA was hybridized on the mouse Immunology NanoString plate and expression was read on an nCounter^®^ Analysis System. Groups compared using limma in R/Bioconductor (see Methods) (JPG 125 kb)

Additional file 3: Figure S3. showing reduced C5a receptor expression and inflammatory responses in V-KO splenic monocytes. (**A**) Splenic monocytes from WT and KO mice were gated on F4/80, Gr1, and Cd11b, and then C5aR-positive cells were counted. F4/80^+^/Gr1^+^/Cd11b^+^/C5aR^+^ splenic monocytes were isolated and then cultured for 24 hours in the presence of LPS. Secreted levels of (**B**) TNF, (**C**) IL-17, and (**D**) IL-12p70 measured by ELISA (JPG 67 kb)

## Abbreviations

APC: Antigen-presenting cells
BMDM: Bone marrow-derived macrophage
CAIA: Collagen antibody-induced arthritis
EAE: Experimental autoimmune encephalomyelitis
IL: Interleukin
Il1rn: IL-1 receptor antagonist
MMP: Matrix metalloproteinase
RA: Rheumatoid arthritis
VISTA: V domain-containing Ig suppressor of T-cell activation
V-KO: VISTA knockout
