# Supplementary figures and images for: VISTA deficiency attenuates antibody-induced arthritis and alters macrophage gene expression in response to simulated immune complexes
Source: Arthritis Res Ther. 2017 Dec 8;19:270. doi: 10.1186/s13075-017-1474-y (PMC5721690; doi:10.1186/s13075-017-1474-y)

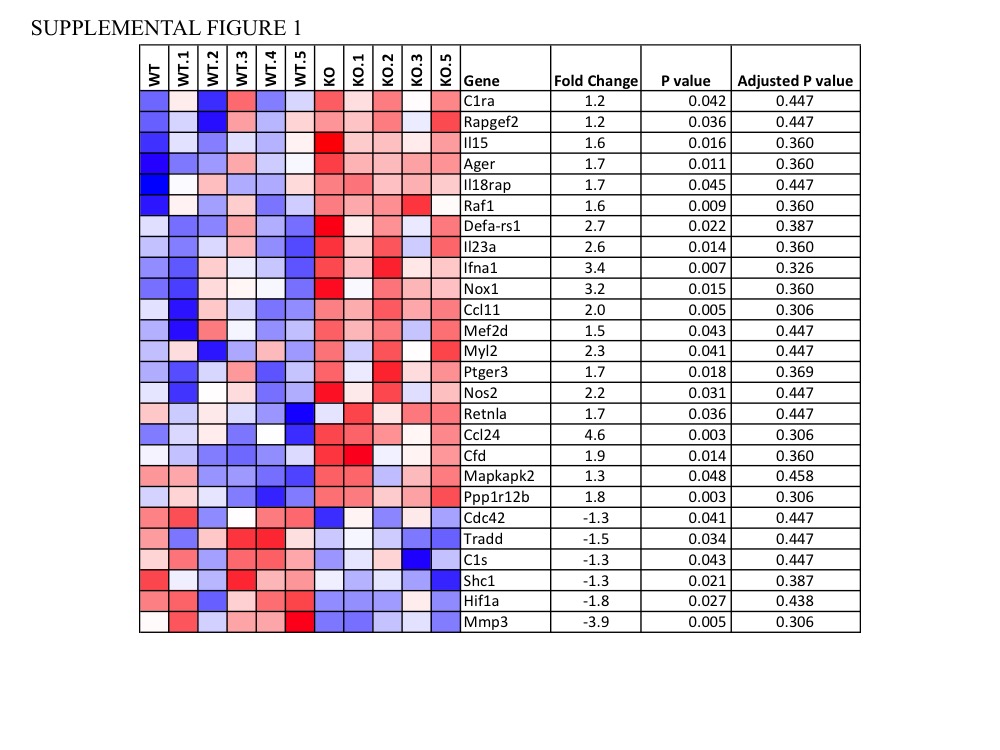

Supplement: Supplementary file 1 — showing gene profile of knee joints from WT and V-KO mice on day 13 of CAIA. RNA isolated from WT (n = 6) and KO (n = 5) paraffin-embedded joints. To examine gene expression, RNA was hybridized on the Mouse Inflammation NanoString plate and expression was read on an nCounter® Analysis System. Each gene compared by unpaired t tests, raw p values presented alongside adjusted p values (see Methods) (JPG 166 kb) [file 13075_2017_1474_MOESM1_ESM.jpg]

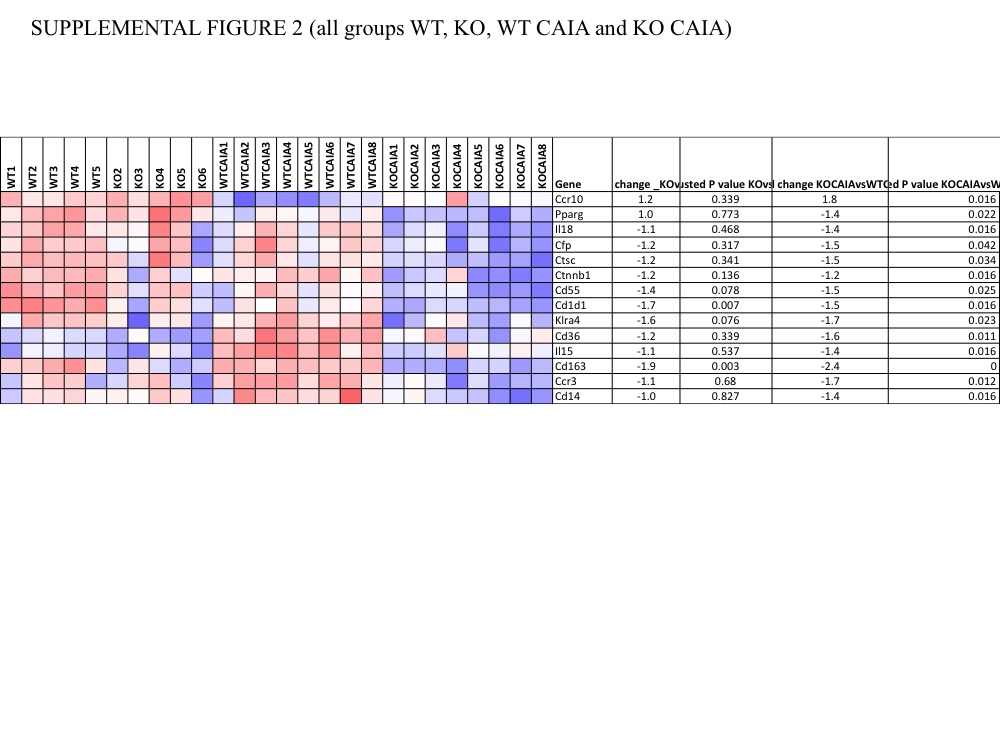

Supplement: Supplementary file 2 — showing comparison of splenic gene expression profiles for arthritic and nonarthritic WT versus V-KO mice. RNA isolated from snap-frozen spleens taken from WT (n = 5), V-KO (n = 5), WT CAIA (n = 8), and V-KO CAIA (n = 8) mouse. To examine gene expression, RNA was hybridized on the mouse Immunology NanoString plate and expression was read on an nCounter® Analysis System. Groups compared using limma in R/Bioconductor (see Methods) (JPG 125 kb) [file 13075_2017_1474_MOESM2_ESM.jpg]

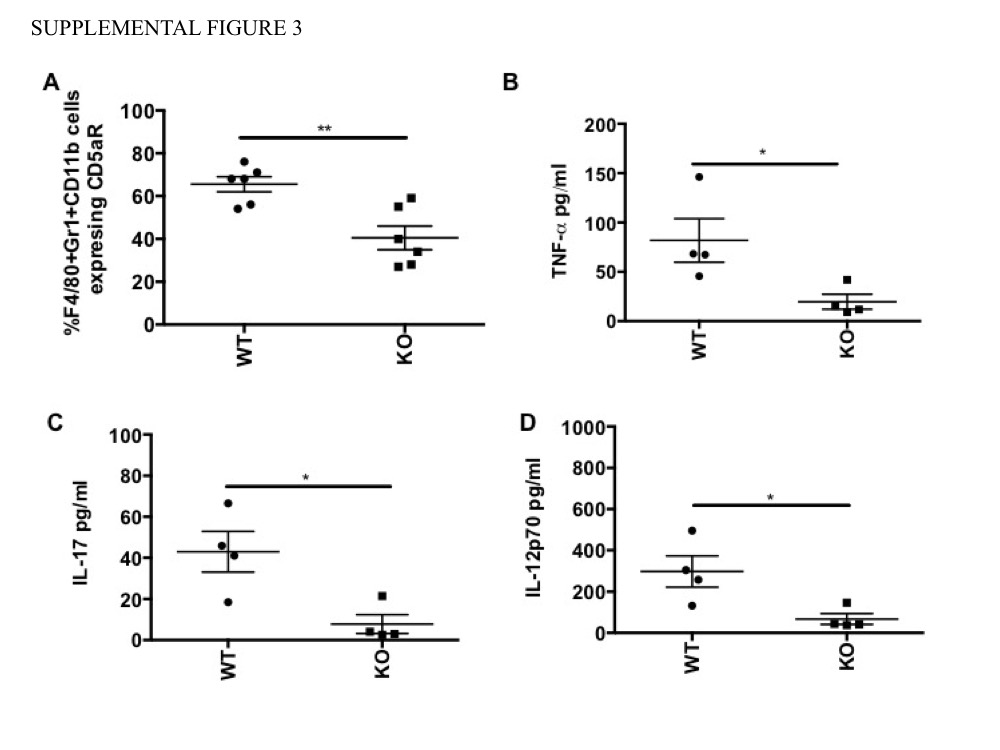

Supplement: Supplementary file 3 — showing reduced C5a receptor expression and inflammatory responses in V-KO splenic monocytes. (A) Splenic monocytes from WT and KO mice were gated on F4/80, Gr1, and Cd11b, and then C5aR-positive cells were counted. F4/80+/Gr1+/Cd11b+/C5aR+ splenic monocytes were isolated and then cultured for 24 hours in the presence of LPS. Secreted levels of (B) TNF, (C) IL-17, and (D) IL-12p70 measured by ELISA (JPG 67 kb) [file 13075_2017_1474_MOESM3_ESM.jpg]
